# Delays in HIV diagnosis and associated factors among patients presenting with advanced disease at a tertiary care hospital in Beijing, China

**DOI:** 10.1371/journal.pone.0182335

**Published:** 2017-08-09

**Authors:** Jing Xie, Evelyn Hsieh, Meng-qing Sun, Huan-ling Wang, Wei Lv, Hong-wei Fan, Tai-sheng Li

**Affiliations:** 1 Department of Infectious Diseases, Peking Union Medical College Hospital, Peking Union Medical College and Chinese Academy of Medical Sciences, Beijing, China; 2 Clinical Immunology Center, Chinese Academy of Medical Sciences, Beijing, China; 3 Section of Rheumatology, Yale School of Medicine, New Haven, Connecticut, United States of America; 4 Department of Surgery, Peking Union Medical College Hospital, Peking Union Medical College and Chinese Academy of Medical Sciences, Beijing, China; Institut Pasteur of Shanghai Chinese Academy of Sciences, CHINA

## Abstract

Delayed diagnosis of HIV infection is associated with advanced immunosuppression and increased risk of onward transmission. Little data exists regarding factors associated with diagnostic delays among patients presenting with advanced HIV disease in China. Medical records of patients with HIV/AIDS hospitalized at a 2000-bed tertiary hospital in Beijing, China between 1997 and 2012 were retrospectively reviewed. Demographic and clinical data of patients newly diagnosed with HIV at the hospital were abstracted. Patient characteristics, disease parameters, and the time interval between the first medical visit and the visit leading to HIV diagnosis were compared among three periods: 1997–2002, 2003–2008 and 2009–2012. Chi-square, Kruskal-Wallis and logistic regression analyses were used as appropriate. A quarter of patients (72/279) were newly diagnosed with HIV at the hospital, consisting of 11, 29 and 32 patients in 1997–2002, 2003–2008 and 2009–2012 respectively. The median time delay between the first medical visit and the visit leading to HIV diagnosis decreased over time from 91 days among patients diagnosed before 2002, to 75 days between 2003 to 2008, and 39 days after 2009 (*p* = 0.036). However, the median CD4^+^T cell count at diagnosis was 26 cells/μL [interquartile range 3–132 cells/μL] in 1997–2002, and remained unchanged across time intervals. Forty-one (57%) patients had AIDS-defining conditions and *Pneumocystis jiroveci* pneumonia was the most common opportunistic infection (31 cases). These results reveal persistent missed opportunities for timely HIV testing among patients with advanced disease. Strategies for promoting early HIV testing in healthcare settings are needed in China.

## Introduction

Delayed HIV diagnosis is associated with advanced immune compromise, increased risk of suboptimal responses to antiretroviral therapy (ART) [1], missed opportunities for preventing onward transmission and increased costs for the healthcare system [2–4]. There is consensus that early diagnosis of HIV infection and entry to care are essential for controlling HIV prevalence. However, a substantial proportion of HIV-infected individuals are still diagnosed at a late stage of disease worldwide. In the US, among persons aged 13 years and older, a quarter of infections were classified as having AIDS at the time of diagnosis [5]. In Europe, 54% of newly diagnosed HIV-infected individuals were late presenters as defined by having CD4^+^T cell counts below 350cells/μL or an AIDS-defining illness within six months of HIV diagnosis [6]. Thus, identifying the factors associated with delayed diagnosis and attempting early testing is of great importance.

Recent studies in developed countries have demonstrated that non-traditional risk groups including women, heterosexual individuals and older adults are more likely to be tested later in the course of HIV infection [7–9]. These data provide important information for policy-making regarding HIV screening strategies in those settings. In China, considerable resources have been invested for HIV testing and care. Parallel systems have been implemented through the Chinese Centre for Disease Control and Prevention (China CDC) and public-sector healthcare facilities to focus on the public health and patient care-related aspects of HIV infection, respectively [10]. According to data from the Chinese national epidemiology database, from January 1, 1985 to December 31, 2009, 41% of newly identified individuals were late presenters at the time of diagnosis [11]. Notably, 30% of new HIV diagnoses were made within the hospital system as opposed to voluntary counselling and testing (VCT) sites of the China CDC system [11]. A study conducted in South China revealed that patients diagnosed in hospital settings have a significantly higher proportion of advanced HIV disease compared with those diagnosed outside of the hospital system [12]. It is likely that these individuals had presented for care at other hospitals or clinics prior to being diagnosed, however little data exist regarding the length of time between first presentation for care and the ultimate date of HIV diagnosis or the clinical characteristics that such patients with advanced disease most commonly present with.

Here, we report a study carried out at a tertiary care hospital in Beijing, China where the vast majority of patients admitted have complex or severe disease requiring multidisciplinary management or surgical intervention, providing a unique opportunity to study the temporal delay from initial presentation for care to eventual HIV diagnosis among patients with advanced disease.

## Methods

### Study design

This retrospective study was conducted from March 2015 to January 2016 at Peking Union Medical College Hospital (PUMCH), a 2000-bed tertiary care center in Beijing, China. The hospital provides medical services to patients from local regions (Beijing, Tianjin and surrounding Hebei province) as well as those referred from other provinces and municipalities of China. All hospitalized HIV/AIDS patients were identified based upon a list generated from the electronic database of the Medical Records Department at PUMCH. Hospital records of these patients were reviewed and eligible participants were selected according to the inclusion criteria of this study. Data from eligible patients were de-identified and entered into an electronic database which was completely anonymous. After data collection was completed, the authors no longer had access to patient identifiers nor the original medical records. The present study was reviewed and approved by the Institutional Review Board (IRB) of PUMCH. The IRB waived the requirement to obtain informed consent from each patient because the study met the IRB’s minimal risk waiver criteria.

### Participants

Patients were eligible for inclusion if they had been hospitalized at PUMCH between January 1, 1997 and December 31, 2012, if their discharge diagnosis included AIDS and/or HIV infection but admitting diagnosis did not, and if HIV seropositive status was identified at PUMCH. Patients referred from outside healthcare settings solely for HIV testing and/or treatment were excluded. HIV diagnosis was based on positive enzyme-linked immunosorbent assay (ELISA) results from two peripheral blood samples and confirmatory western blot assay in accordance with the guidelines issued by the China CDC. Because the National Free Antiretroviral Treatment Program (NFATP) was piloted in 2002 and scaled up nation-wide in 2003, and PUMCH was designated as a NFATP provider at the end of 2008, patients were divided into three groups according to their time of diagnosis: 1997–2002, 2003–2008, and 2009–2012.

### Data collection

All identifiable health care-related visits, including outside medical visits and PUMCH visits, were abstracted from the history of present illness of their hospital records. Visits were divided into two categories as defined previously by Kuo et al: diagnostic health care visits (DHVs), which were visits at PUMCH that directly led to the HIV diagnosis, and previous health care visits (PHVs), which included all other documented inpatient or outpatient medical visits for related symptoms prior to the DHVs [13]. The dates of the DHV and first PHV were recorded for each patient and the length of time between the first PHV and the DHV was calculated. For patients who had PHVs at PUMCH prior to their DHVs, the date of the first PUMCH PHV was also recorded.

Patient demographics at the time of diagnosis including age, gender, place of residence and occupation, were abstracted. Variables collected from the history of present illness included any of the following symptoms or events: fever, unexplained weight loss, recurrent or chronic diarrhea, recurrent pneumonia, oral candidiasis, vision impairment, and varicella zoster infection. Physical examination findings collected from the DHVs included fever (≥ 37.3°C), thrush, generalized lymphadenopathy, and hepatosplenomegaly. Laboratory data collected included presence of anemia (hemoglobin ≤ 120 g/L for men, ≤ 110 g/L for women), leukopenia (white blood cell count ≤ 4 000 cells/**μ**L), lymphopenia (< 1 000 cells/**μ**L), and thrombocytopenia (platelet count ≤ 100 000 /**μ**L). Hepatitis B surface antigen (HBsAg) and hepatitis C virus antibody (anti-HCV), CD4^+^T cell count and plasma HIV RNA level at the time of diagnosis were also abstracted from the medical records.

### Statistical analysis

Descriptive analyses of patient characteristics at the time of diagnosis were performed. Continuous variables were expressed as medians (interquartile range, IQR) and compared across the three time intervals using the Kruskal-Wallis *H* Test. Categorical variables were expressed as frequencies and compared using the Chi-square or Fisher’s exact test, as appropriate. To identify factors associated with prolonged diagnostic delay from the time of the first PHV to the DHV—defined as >63 days (the observed median value)—separate univariate logistic regression models were used to assess the odds ratio (OR) for each candidate variable, including gender, place of residence, occupation, time interval, CD4^+^T cell count, number of symptoms/events documented in the history of present illness, number of findings on physical examination, and presence of an AIDS-defining illness. Multivariable logistic regression models were then developed using variables with *p*-value < 0.15 in the univariate models. Backward stepwise elimination was used to fit the multivariable models. Statistical analysis was performed using SPSS 20.0 (IBM Corporation, Armonk, New York, USA) and GraphPad Prism 6.0 (GraphPad Software, Inc. La Jolla, CA, USA). A *p*-value < 0.05 was considered statistically significant.

## Results

### Demographics and HIV-associated parameters

A total of 279 patients with HIV/AIDS were hospitalized during the study period. Of these, 72 (26%) patients—consisting of 69 adults and three pediatric patients (3, 8 and 9 years old)—were newly diagnosed at PUMCH. There were 11, 29 and 32 patients diagnosed during 1997–2002, 2003–2008 and 2009–2012, respectively. The majority of these patients were male and 68% were between 30–49 years of age (Table 1). Nearly two-thirds of patients were from other provinces across China. Manual laborer/farmer was the most commonly reported occupation in 1997–2002, whereas professional, manager or administrative work was most commonly reported in 2009–2012. The proportion of patients diagnosed in the Emergency Department increased from 0% in 1997–2002 to 24% in 2003–2008, and then to 38% in 2009–2012. The median hospitalization time increased from 14 days in 1997–2002 to 29 days in 2009–2012. For those whom route of transmission was determined, blood-borne transmission was predominant in 1997–2002 (55%) and 2003–2008 (45%), whereas sexual transmission was most commonly reported in 2009–2012 (41%).

**Table 1:** Demographic characteristics and HIV-related parameters of patients at the time of HIV diagnosis.

	Overall	1997–2002	2003–2008	2009–2012	p*
Number	72	11	29	32	
Male gender *n(%)*	53(74)	7(64)	18(62)	28(88)	0.05
Age *median years (IQR)*	41(32–46)	31(29–39)	42(32–47)	41(33–47)	0.05
Place of residence *n(%)*					0.64
Local (Beijing, Tianjin and surrounding Hebei Provinc*)	27(38)	3(27)	10(35)	14(44)	
Other regions across China**	45(63)	8(73)	19(66)	18(56)	
Occupation *n(%)*					<0.001
Child/student	3(4)	0(0)	3(10)	0(0)	
Unemployed	7(10)	1(9)	5(17)	1(3)	
Manual laborer/farmer	28(39)	8(73)	14(48)	6(19)	
Professional, managerial, or administrative work	26(36)	2(18)	6(21)	18(56)	
Missing data	8(11)	0(0)	1(3)	7(22)	
Clinical department where diagnosis was made *n(%)*					0.04
Emergency department	19(26)	0(0)	7(24)	12(38)	
Other departments***	53(74)	11(100)	22(76)	20(63)	
Length of hospitalization *median days (IQR)*	26(14–42)	14(7–26)	27(12–48)	29(18–42)	0.04
Transmission *n(%)*					0.001
Blood-borne	21(29)	6(55)	13(45)	2(6)	
Male-to-male	12(17)	0(0)	3(10)	9(28)	
Heterosexual	9(13)	2(18)	3(10)	4(13)	
Mother-to-child	3(4)	0(0)	3(10)	0(0)	
Unknown	27(38)	3(27)	7(24)	17(53)	
CD4^+^T cell count (n = 71) *median cells/μL (IQR)*	24(6–101)	26(3–132)	27(5–88)	22(6–143)	0.96
CD4^+^T cell count *n(%)*					0.93
≤ 50 cells/μL	46(65)	8(73)	18(64)	20(63)	
51–100 cells/μL	7(10)	0(0)	4(14)	3(9)	
101–200 cells/μL	9(13)	2(18)	3(11)	4(13)	
> 200 cells/μL	9(13)	1(9)	3(11)	5(16)	
HIV RNA (n = 49) *median log10 copies/mL (IQR)*	5.08(4.51–5.46)	5.08(4.47–5.62)	5.14(4.40–5.66)	4.96(4.54–5.37)	0.94
Number of patients with AIDS-defining conditions *n(%)*	41(57)	7(64)	17(59)	17(53)	0.85
Total cases of AIDS-defining conditions *n* ****	47	9	19	19	0.77
*Pneumocystis jiroveci* pneumonia *n(%)*	31(66)	5(56)	13(68)	13(68)	
Active tuberculosis *n(%)*	7(15)	3(33)	2(11)	2(11)	
Cytomegalovirus retinitis *n(%)*	5(11)	1(11)	2(11)	2(11)	
Esophageal candidiasis *n(%)*	3(6)	0(0)	2(11)	1(5)	
Pulmonary candidiasis *n(%)*	1(2)	0(0)	0(0)	1(5)	

IQR, interquartile range; PUMCH, Peking Union Medical College Hospital; HIV, human immunodeficiency virus; AIDS, acquired immune deficiency syndrome

*p values are for comparisons among the three time intervals.

**Regional distribution of patients from other provinces across China from the highest to the lowest was: the North (15), the West (11), the Central (10), the East (8), and lastly, the South region (1).

***Numbers of patients diagnosed in other departments from the highest to the lowest were: Infectious Diseases (20), Pulmonology and Neurology (8 for each department), General Internal Medicine (5), Rheumatology (3), Pediatrics, Endocrinology and Gastroenterology (2 for each department), Ophthalmology, Oncology, and Traditional Chinese Medicine (1 for each department).

****Six patients had *Pneumocystis jiroveci* pneumonia plus other opportunistic infections (three with active tuberculosis, two with esophageal candidiasis, and one with pulmonary candidiasis).

CD4^+^T cell counts were available for 71 (99%) patients at diagnosis. The median CD4^+^T cell count was only 24 (IQR 6–101) cells/μL (Table 1). Nearly two-thirds of patients (46/71, 65%) had a CD4^+^T cell count below 50 cells/**μ**L. Only 9 (13%) patients had a CD4^+^T cell count above 200 cells/**μ**L. Initial plasma HIV RNA levels were available for 49 (68%) patients with a median value of 5.08 (IQR 4.51–5.46) log10 copies/mL. Neither CD4^+^T cell counts nor HIV RNA levels differed across the three time periods. More than half of patients (41/72, 56.9%) had at least one AIDS-defining condition. *Pneumocystis jiroveci* pneumonia (PCP) was the most common opportunistic infection (31 cases), followed by active tuberculosis (7 cases), cytomegalovirus retinitis (5 cases), esophageal candidiasis (3 cases) and pulmonary candidiasis (1 case). The distribution of AIDS-defining conditions was also similar among the three groups. Patients with AIDS-defining conditions had significantly lower median CD4^+^T cell counts [12 (4–52) cells/μL] compared to those without [49 (17–196) cells/μL, *p* = 0.003]. Among patients with AIDS-defining conditions, most documented diagnoses during their PHVs were community-acquired pneumonia (28 cases), whereas for patients without AIDS-defining conditions, 15 (48%) had an undetermined diagnosis during their PHVs.

### Symptoms and signs prior to diagnosis with HIV

Among the events in each patient’s history of present illness, fever was the most common symptom (85%) followed by unexplained weight loss (68%), recurrent or chronic diarrhea (21%), oral candidiasis (18%), recurrent pneumonia (13%), varicella zoster infection (10%), and lastly, vision impairment (8%) (Table 2). Among the physical examination findings at the time of the DHV, fever was most common (69%) followed by oral thrush (33%), generalized lymphadenopathy (21%) and hepatosplenomegaly (10%). Frequencies of these symptoms and signs were comparable among the three groups. Laboratory evaluations showed that 41 (57%) patients had lymphopenia, 26 (36%) had anemia, 20 (28%) had leucopenia, and 7 (10%) had thrombocytopenia. Anti-HCV and HBsAg was positive among 11 (17%) and 3 (4%) patients, respectively. The anti-HCV seropositivity rate decreased significantly over time from 44% in 1997–2002 to 0 in 2009–2012 (*p* < 0.001).

**Table 2:** Symptoms and events in patients’ history of present illness, signs and laboratory findings at the time of diagnosis.

	Overall N = 72	1997–2002 N = 11	2003–2008 N = 29	2009–2012 N = 32
Symptoms/events in history of present illness *n(%)*				
Fever	61(85)	9(82)	23(79)	29(91)
Unexplained weight loss	49(68)	9(82)	17(59)	23(72)
Recurrent or chronic diarrhea	15(21)	4(36)	3(10)	8(25)
Oral candidiasis	13(18)	3(27)	4(14)	6(19)
Recurrent pneumonia	9(13)	2(18)	2(7)	5(16)
Varicella zoster infection	7(10)	1(9)	2(7)	4(13)
Vision impairment	6(8)	1(9)	3(10)	2(6)
Physical examination findings *n(%)*				
Fever	50(69)	9(82)	16(55)	25(78)
Oral thrush	24(33)	5(46)	9(31)	10(31)
Generalized lymphadenopathy	15(21)	3(27)	3(10)	9(28)
Hepatosplenomegaly	7(10)	3(27)	2(7)	2(6)
Laboratory findings *n(%)*				
Lymphocyte count < 1 000 cells/μL	41(57)	5(46)	17(59)	19(59)
Hemoglobin < 12 (men) or 11 g/dL (women)	26(36)	3(27)	11(38)	12(38)
White blood cell count < 4 000 cells/μL	20(28)	6(55)	4(14)	10(31)
Platelet count < 100 000 cells/μL	7(10)	1(9)	3(10)	3(9)
Anti-HCV seropositivity*	11(17)	4(44)	7(29)	0(0)
HBsAg seropositivity**	3(4)	1(10)	1(4)	1(3)

Anti-HCV, hepatitis C virus antibody; HBsAg, hepatitis B surface antigen.

*Anti-HCV was tested among 9, 24 and 32 patients diagnosed during 1997–2002, 2003–2008 and 2009–2012, respectively.

**HBsAg was tested among 10, 27, and 32 patients diagnosed during 1997–2002, 2003–2008 and 2009–2012, respectively.

### Delay in time from the first PHV to DHV and associated factors

The overall median delay from the first PHV to DHV was 63 (IQR 26–135) days (Table 3). Of the 72 patients included in the study, 71 (99%) patients had their first PHVs at medical facilities outside of PUMCH and none of them were offered HIV testing during those outside medical visits. The median time interval from the first PHV to the first PUMCH visit was 58 (IQR 26–135) days and 0 (IQR 0–4) days from the first PUMCH visit to the DHV. The median time interval from the first PHV to the DHV decreased over time with a delay of 91 days in patients diagnosed before 2002, followed by 75 days between 2003 and 2008 and 39 days after 2009 (*p* = 0.036). Similar results were seen with the time interval from the first PHV to the first PUMCH visit: 88 days before 2002, 75 days between 2003 to 2008, and 34 days after 2009 (*p* = 0.047). No significant difference was found in the time interval from the first PHMCH visit to DHV among the three period groups.

**Table 3:** Time delay from the first PHV to DHV.

Time interval (Days)	Overall	1997–2002	2003–2008	2009–2012	p*
First PHV to DHV	63(26–135)	91(58–161)	75(33–139)	39(15–104)	0.036
First PHV to first PUMCH visit	58(26–135)	88(58–158)	75(31–139)	34(12–101)	0.047
First PUMCH visit to DHV	0(0–4)	3(0–20)	0(0–4)	0(0–0)	0.084

PHV, previous health care visits; DHV, diagnostic health care visits, PUMCH, Peking Union Medical College Hospital.

*p values are for comparison among the three period groups.

Multivariable analysis demonstrated that being diagnosed in 1997–2002 (OR 6.35, 95% CI 1.15–34.90, *p* = 0.03) or 2003–2008 (OR 5.36, 95% CI 1.45–19.85, *p* = 0.01) was independently associated with prolonged time delay from the first PHV to DHV (Table 4). Presentation with > two symptoms or events in the history of present illness (OR 7.16, 95% CI 2.00–25.60, *p* = 0.002) was also significantly associated with prolonged diagnostic time delay. Prolonged time delay was not found to be significantly associated with other factors including place of residence, occupation, gender, CD4^+^T cell count, number of findings on physical examination at the DHV, and presence of an AIDS-defining condition.

**Table 4:** Factors associated with prolonged delay in time from the first PHV to DHV.

	Univariate		Multivariable	
	OR(95% CI)	*p*	OR(95% CI)	*p*
Gender				
Female	Reference			
Male	0.65(0.23–1.87)	0.42		
Place of residence				
Beijing, Tianjin and surrounding Hebei Province	Reference		Reference	
Other provinces across China	2.33(0.87–6.20)	0.09	1.83(0.59–5.71)	0.30
Occupation				
Professional, managerial, or administrative work	Reference			
Others	1.79(0.65–4.91)	0.26		
Time period				
2009–2012	Reference		Reference	
2003–2008	2.71(0.96–7.64)	0.06	5.36(1.45–19.85)	0.01
1997–2002	5.09(1.12–23.14)	0.04	6.35(1.15–34.90)	0.03
CD4^+^T cell count				
> 50 cells/μL	Reference		Reference	
≤ 50 cells/μL	2.31(0.85–6.30)	0.10	2.55(0.81–8.03)	0.11
Number of symptoms/events in the history of present illness				
≤ 2 (the observed median)	Reference		Reference	
> 2	3.91(1.41–10.88)	0.01	7.16(2.00–25.60)	0.002
Number of findings in the physical examination				
≤ 1 (the observed median)	Reference			
> 1	0.63(0.24–1.62)	0.34		
Presence of an AIDS-defining condition				
No	Reference			
Yes	0.89(0.35–2.27)	0.81		

OR, odds ratio; CI, confidence interval; AIDS, acquired immune deficiency syndrome.

## Discussion

This study conducted at a large tertiary care hospital in Beijing, China is the first, to our knowledge, to quantify the delay in time from initial presentation to the health care system and ultimate HIV diagnosis in patients with advanced HIV/AIDS. Our data show that although the length of diagnostic delay period declined from 91 days in 1997–2002 to 39 days in 2009–2012, the severity of disease at the time of diagnosis remained unchanged over time, as demonstrated by the fact that > 60% of patients had CD4^+^T cell counts below 50 cells/μL across all the three time periods. We also found that although a large proportion of patients had clues for immune compromise, they had not been offered HIV testing during previous medical visits. These results identify missed opportunities for more timely HIV testing in medical settings, which have clinical consequences [1, 14] and can negatively impact overall public health [2–4].

As of the end of 2014, 295 358 patients in China were receiving ART through the NFATP and 96.5% of the counties/districts across the country had qualified HIV testing labs within various medical and healthcare facilities [15]. Although the threshold CD4^+^T cell count for ART initiation in the NFATP was raised from 200 cells/μL in 2002 to 350 cells/μL in 2008, over 50% of patients initiating ART between 2010 and 2011 still had a pretreatment CD4^+^T cell count below 200 cells/μL [16]. Another study demonstrated that delayed ART and pretreatment CD4^+^T cell count < 50 cells/μL were the strongest risk factors for overall HIV-related mortality among adults in the NFATP [11]. Taken together, an urgent need exists for earlier HIV diagnosis, both to prevent individual morbidity and mortality from severe immunosuppression, and to better control HIV transmission in China.

One reason underlying missed opportunities for earlier diagnosis may be a lack of routine HIV screening by clinicians when patients present for medical care, even when associated symptoms are present. Since 2003, client-initiated VCT has been the primary approach to HIV screening in China. In 2007 the World Health Organization (WHO) began recommending provider-initiated HIV testing and counseling (PITC) strategies to expand access to HIV-related services [17]. Subsequently, studies in China have demonstrated that PITC is well accepted among tuberculosis patients (99%) [18] and male attendees of sexually transmitted diseases (STD) clinics in China (91%) [19]. However, a survey of six public STD clinics in South China demonstrated that in practice, physicians providing STD care offered HIV testing to only 28% of their patients [20]. Low perceived HIV prevalence and absence of a formal recommendation in current guidelines were the most common physician-reported barriers to offering HIV testing [20]. These results highlight the under-recognition of HIV infection by clinicians in primary care settings in China, even among higher risk populations, and the need for efforts to increase awareness and encourage screening. Compared with VCT, PITC strategies rely on providers to offer testing. Therefore, more research about clinician attitudes, behaviors and practices is essential in order to identify feasible PITC strategies to reduce barriers to testing in healthcare facilities.

Furthermore, over the study period, there has been a notable shift in prevalent HIV genotypes in China. The proportion of circulating recombinant strain (CRF) 01_AE has increased significantly, especially in the population of patients acquiring HIV through sexual transmission, and CRF01_AE has now become the most prevalent genotype since 2006 [21]. Studies have shown that compared with previous predominant subtypes (for example, CRF07_BC and CRF08_BC) the CRF01_AE strain is associated with more rapid declines in CD4^+^T cell counts [22, 23]. This biological characteristic of the virus might explain, in part, the persistently low CD4^+^T cell counts observed among our patients over time despite the decrease in length of diagnostic delay, and further underscores the importance of strategies to promote timely HIV testing in clinical settings.

Several limitations of this study warrant mention. First, due to resource constraints, our study sample was limited to patients admitted at a single tertiary care hospital in Beijing, and did not include patients from other tertiary hospitals. Therefore, the volume of patients newly diagnosed with HIV during the study period was not large. However, it is notable that 63% of the patients included in our study were from outside of the Beijing/Tianjin/Hebei region. Second, patients diagnosed in the outpatient clinic at PUMCH were not included because detailed outpatient medical records were not available across all three time periods. As a result, our sample represents more complex and advanced cases of HIV and cannot be generalized to patients with asymptomatic or mild HIV infection. Nevertheless, over the study period we found participants were increasingly from urban centers, were more likely to acquire HIV through sexual modes of transmission, and had decreasing rates of Hepatitis C co-infection. These key epidemiologic features are consistent with national HIV trends [15]. Therefore, despite the limitations of our study, we believe our overarching findings underscore the diagnostic challenges that remain among individuals with advanced HIV in China.

## Conclusions

Temporal delays in diagnosis among patients with advanced HIV have improved significantly over a 15-year period. In this study, we identified persistent missed opportunities for timely HIV testing in healthcare settings despite increasing general awareness of HIV among patients and providers, and increased availability of HIV-related services over the past decade. Targeted strategies for promoting uptake of early HIV testing practices in healthcare settings are imperative to change this phenomenon in China, and may benefit from an increased emphasis on provider-initiated screening. Future studies should also focus on identifying pragmatic factors influencing the successful implementation and uptake of such screening strategies including feasibility, patient and provider acceptability, and provider prioritization.

## Supporting information

S1 File The data set of this study. The participant-level data that underlies the results of this study.
(XLSX)

S2 File The STROBE checklist. (DOCX)
